# Tripartite Motif-Containing Protein 32 (TRIM32): What Does It Do for Skeletal Muscle?

**DOI:** 10.3390/cells12162104

**Published:** 2023-08-19

**Authors:** Seung Yeon Jeong, Jun Hee Choi, Jooho Kim, Jin Seok Woo, Eun Hui Lee

**Affiliations:** 1 Department of Physiology, College of Medicine, The Catholic University of Korea, Seoul 06591, Republic of Korea; 2 Department of Biomedicine & Health Sciences, Graduate School, The Catholic University of Korea, Seoul 06591, Republic of Korea; 3 Department of Physiology, David Geffen School of Medicine, University of California Los Angeles, Los Angeles, CA 10833, USA

**Keywords:** TRIM32, NHL repeats, muscular dystrophy, LGMD2H, DMD

## Abstract

Tripartite motif-containing protein 32 (TRIM32) is a member of the tripartite motif family and is highly conserved from flies to humans. Via its E3 ubiquitin ligase activity, TRIM32 mediates and regulates many physiological and pathophysiological processes, such as growth, differentiation, muscle regeneration, immunity, and carcinogenesis. TRIM32 plays multifunctional roles in the maintenance of skeletal muscle. Genetic variations in the *TRIM32* gene are associated with skeletal muscular dystrophies in humans, including limb–girdle muscular dystrophy type 2H (LGMD2H). LGMD2H-causing genetic variations of TRIM32 occur most frequently in the C-terminal NHL (ncl-1, HT2A, and lin-41) repeats of TRIM32. LGMD2H is characterized by skeletal muscle dystrophy, myopathy, and atrophy. Surprisingly, most patients with LGMD2H show minimal or no dysfunction in other tissues or organs, despite the broad expression of TRIM32 in various tissues. This suggests more prominent roles for TRIM32 in skeletal muscle than in other tissues or organs. This review is focused on understanding the physiological roles of TRIM32 in skeletal muscle, the pathophysiological mechanisms mediated by TRIM32 genetic variants in LGMD2H patients, and the correlations between TRIM32 and Duchenne muscular dystrophy (DMD).

## 1. Introduction

More than six hundred skeletal muscles exist in the human body, and maintain body posture and movements [1]. Depending on age, sex, daily physical activity, or illness of an individual, skeletal muscle constitutes approximately 40–50% of the total body weight and expresses approximately 50–70% of all the proteins in the human body. To maintain homeostasis in terms of skeletal function and mass, skeletal muscle fibers (i.e., skeletal muscle cells) respond to various physiological and pathophysiological stimuli from internal and external muscle fibers.

Tripartite motif-containing protein 32 (TRIM32) was discovered via yeast two-hybrid analysis in 1995 as a protein that interacts with Tat, a key trans-activator of the viral transcription of HIV-1 [2]. TRIM32 has E3 ubiquitin ligase activity and is expressed in a variety of tissues in humans, including all three types of muscle [3]. Via its E3 ubiquitin ligase activity, TRIM32 mediates and regulates physiological and pathophysiological processes, such as growth, differentiation, muscle regeneration, immunity, carcinogenesis, and other cellular processes [4].

With special attention to muscle maintenance in terms of regeneration and atrophy, this review is focused on the roles of TRIM32 and its E3 ubiquitin ligase activity in skeletal muscle; specifically, TRIM32-interacting proteins in skeletal muscle, expression levels of TRIM32 under various conditions, such as cellular senescence and terminal differentiation, and patients with muscular dystrophies and atrophies due to LGMD2H. In addition, genetic variants of TRIM32, animal models of LGMD2H, and correlations between TRIM32 and Duchenne muscular dystrophy (DMD) are described.

## 2. Skeletal Muscle

### 2.1. Regenerative Skeletal Muscle

Skeletal muscle is striated and is characterized by two remarkable properties not found in other tissues or organs [5,6]. Skeletal muscle cells contain multiple nuclei due to the fusion of cells with a single nucleus, which makes skeletal muscle cells very long; for example, human sartorius muscle is approximately 60 cm long in tall individuals [5,6,7]. This is why skeletal muscle cells are also called “muscle fibers”. In contrast to other tissues in adults, skeletal muscle can regenerate in response to injuries by activating quiescent satellite cells (i.e., skeletal muscle stem cells in adult skeletal muscle) [6]. The fusion of active satellite cells (also called primary myoblasts under in vitro culture conditions) with existing muscle fibers results in the formation of longer and thicker muscle fibers, which regenerate skeletal muscle at injury sites. The process of longer and thicker muscle fiber formation is called terminal differentiation, which must be distinguished from the differentiation of proliferating satellite cells during development.

### 2.2. Types of Skeletal Muscle Fibers

Skeletal muscle produces energy mainly through glycogen and fatty acid metabolism, and vertebral skeletal muscle fibers are divided into three types based on their ability to produce ATP and shortening velocity [8,9,10,11]. Slow oxidative fibers (slow-twitch or type I fibers) with slow contractile speeds produce ATP via aerobic respiration involving fatty acids. Fast oxidative fibers (type IIa or red fibers) produce ATP through both aerobic respiration and anaerobic glycolysis and are more prone to fatigue than slow oxidative fibers. Fast glycolytic fibers (fast-twitch or type IIb fibers) with faster contractile speeds than fast oxidative fibers produce ATP through anaerobic glycolysis involving glycogen and a relatively small amount of glucose.

In addition to the production and use of ATP, skeletal muscle is related to metabolic proteins, and alterations in metabolism are related to skeletal muscle dystrophies, and vice versa [12,13,14,15,16]. In mammalian striated muscle, including skeletal muscle, some glycolytic enzymes are located at the M-line and Z-line of a sarcomere (the smallest contractile unit in striated muscle) [14,15,16]. For more details, see the latter part of this review (Section 8.2).

### 2.3. Contraction and Relaxation of Skeletal Muscle

Skeletal muscle contracts or relaxes to maintain or change body posture when standing still or during movement. For the contraction of skeletal muscle, the Ca^2+^ level in the cytosol of muscle fibers is temporally and spatially regulated because a several-micromolar concentration of Ca^2+^ in the cytosol functions as a switch to activate a series of contractile proteins by binding to troponin C [5,17]. The cytosolic Ca^2+^ involved in skeletal muscle contraction is supplied by two different Ca^2+^ pools: Ca^2+^ ions that are stored in the sarcoplasmic reticulum (SR) and Ca^2+^ ions in the extracellular space [18,19].

The depolarization of the transverse (t)-tubule membrane in skeletal muscle fibers induces skeletal muscle contraction via various steps, which are collectively called excitation–contraction (EC) coupling [19,20,21,22]. During EC coupling, membrane excitation activates the dihydropyridine receptor (DHPR) on the t-tubule membrane, which in turn activates ryanodine receptor type 1 (RyR1, an internal Ca^2+^ channel) on the SR membrane mediated via the physical interaction between activated DHPR and RyR1. Activated RyR1 allows the cytosolic Ca^2+^ level to be increased from nM to µM levels by releasing Ca^2+^ from the SR into the cytosol [23]. The increase in the cytosolic Ca^2+^ level, which is needed for skeletal muscle contraction, is also achieved via extracellular Ca^2+^ entry into the cytosol. Store-operated Ca^2+^ entry (SOCE) contributes to extracellular Ca^2+^ entry [19,21,24]. SOCE in skeletal muscle fibers is mediated mainly by Orai1 (a Ca^2+^ entry channel on the plasma/t-tubule membrane) and stromal interaction molecule 1 (STIM1, a Ca^2+^ sensor on the SR membrane). In addition to Orai1, canonical-type transient receptor potential cation channels (TRPCs, Ca^2+^ entry channels on the plasma/t-tubule membrane) mediate extracellular Ca^2+^ entry into the cytosol via the SOCE mechanism in skeletal muscle [19,22].

For the relaxation of skeletal muscle, the high cytosolic Ca^2+^ level that is needed for skeletal muscle contraction needs to be returned to the resting level [17,24,25]. Ca^2+^ pumps return cytosolic Ca^2+^ at high levels into the SR or extracellular space. The main Ca^2+^ pumps are sarcoplasmic/endoplasmic reticulum Ca^2+^-ATPase 1a (SERCA1a) in the SR membrane, Na^+^/Ca^2+^ exchanger in the plasma/t-tubule membrane, and Ca^2+^-ATPase in the plasma membrane (called plasma membrane Ca^2+^-ATPase, PMCA).

Costameres in striated muscle cells are subsarcolemmal assemblies of the dystrophin–glycoprotein complex and the integrin–vinculin–talin complex [26,27,28]. By connecting the Z-line of a sarcomere to the plasma membrane, costameres are crucial for the transmission of the sarcomere-generated contractile forces to the plasma membrane and the extracellular matrix [28,29]. In addition, costameres protect the plasma membrane from mechanical stresses during muscle contraction and stretching and maintain plasma membrane alignment with the sarcomere during muscle contraction and relaxation [29,30]. Dysfunctional costameric proteins contribute to skeletal muscular dystrophies, such as DMD [29,30].

## 3. Tripartite Motif-Containing Protein 32 (TRIM32)

### 3.1. TRIM Family

TRIM family proteins are characterized by the N-terminal TRIM motif, including a really interesting new gene (RING) domain, one or two B-box domains, a coiled-coil region, and C-terminal variable regions [31,32]. The human TRIM family consists of more than 80 proteins and contributes to various physiological processes, including protein quality control, apoptosis, development, innate immunity, and others [33]. Alterations in TRIM proteins are involved in pathological processes such as cancers and diseases related to the immune response, development, metabolism, and muscle [34].

Based on the C-terminal variable regions, TRIM proteins are subdivided into twelve subfamilies [32]. Among these families, the C-VII subfamily includes proteins with five or six NHL (named after ncl-1, HT2A, and lin-41) repeats, and the TRIM proteins with NHL repeats regulate cell growth, proliferation, and differentiation via protein-protein interactions via these NHL repeats [35,36,37].

### 3.2. TRIM32 and Its Domains

TRIM32 is a member of the C-VII subfamily in the tripartite motif family and is ubiquitously expressed, including all three types of muscle [3]. TRIM32 is also known as HT2A or TATIP in the literature (GenBank accession code, NM_012210.3; HGNC ID, 16380; Ensembl ID, ENSG00000119401; OMIM ID, 602290; UniProtKB/Swiss-Prot ID, Q13049). Nucleotide and protein sequences of TRIM32 in mice and humans share 87% and 95% identity, respectively [38], suggesting the conservation of TRIM32 across species. TRIM32 is a multifunctional protein that mediates and regulates physiological and pathophysiological processes, such as growth by regulating the cell cycle and cell motility, differentiation, muscle regeneration, immunity, carcinogenesis, and other cellular functions [4].

**Figure 1:**
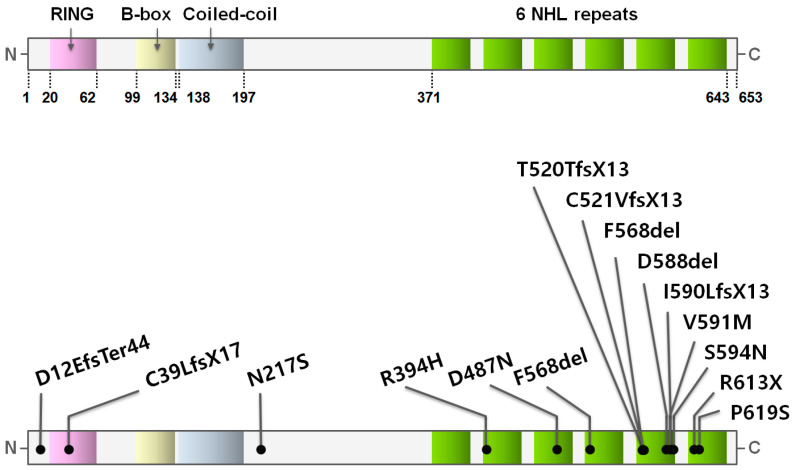
Locations of TRIM32 genetic variants that cause LGMD2H in humans. A schematic diagram showing human TRIM32. Locations of TRIM32 genetic variants that cause LGMD2H in humans and are discussed in this review are presented in the schematic diagram of human TRIM32. Numbers indicate the amino acid position in the sequence. N and C indicate the N-terminus and C-terminus of TRIM32, respectively.

TRIM32 is composed of the RING domain, a type-2 B-box domain, and a coiled-coil region of the typical TRIM motif and six C-terminal NHL repeats (Figure 1) [31]. The RING domain shows E3 ubiquitin ligase activity during the ubiquitination of target proteins. Two zinc ions that are coordinated by cysteine and histidine residues and a proline residue downstream of a cysteine residue at the seventh position of the RING domain provide the structural integrity and enable the E3 ubiquitin ligase activity of TRIM32 [39,40,41,42]. Dimerization of the RING domain promotes the TRIM32 association with E2-ubiquitin, which enhances the transfer of ubiquitin to target proteins [43]. The RING domain and coiled-coil region are involved in the oligomerization of TRIM32, which is required for the E3 ubiquitin ligase activity of TRIM32 [43,44,45].

Similar to the RING domain, the B-box domain coordinates two zinc ions and carries a short α-helix followed by two short β-strands that are separated by a type-2 β-turn [46]. In contrast to the RING domain, the B-box domain is not involved in the oligomerization or E3 ubiquitin ligase activity of TRIM32 but modulates the rate of E3 ubiquitin ligase activity and subcellular localization of TRIM32 [44].

**Figure 2:**
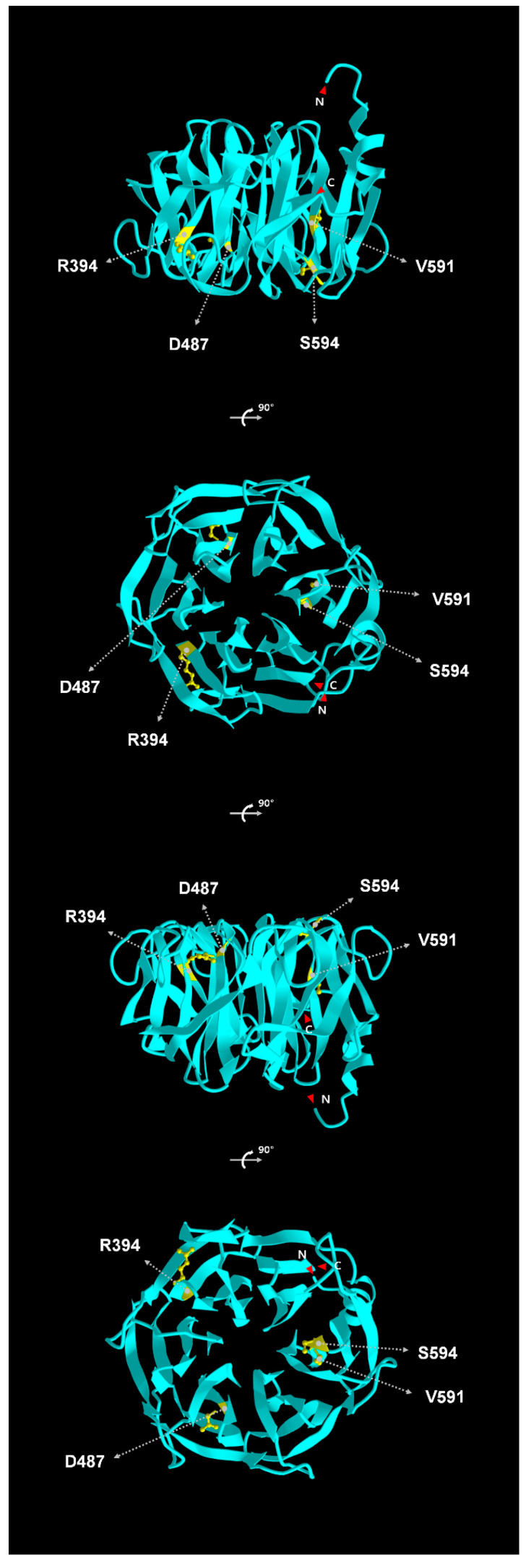
Three-dimensional structure of the NHL repeats in *Drosophila* Abba/Thin. From four different angles via horizontal rotation, the three-dimensional structure of the NHL repeats in *Drosophila* Abba/Thin (PDB ID: 6D69) are presented as a ribbon diagram generated using iCn3D, the NCBI web-based 3D structure viewer. N and C indicate the N-terminus and C-terminus of the NHL repeats, respectively. *Drosophila* Abba/Thin is the *Drosophila* ortholog of TRIM32 [48,49]. Locations of the residues corresponding to four LGMD2H-causing genetic variations in the NHL repeats of human TRIM32 are colored yellow (R394, D487, V591, or S594).

Repeats of the conserved NHL motif are carried by proteins in various prokaryotic and eukaryotic cells [3,47]. The three-dimensional structure of the NHL repeats in *Drosophila* Abba/Thin (the *Drosophila* ortholog of TRIM32; *thin* is the allelic counterpart of *abba* [48,49]) consists of a six-bladed β-propeller in which each NHL repeat forms four antiparallel β-sheets arranged around a central axis (Figure 2) [50]. This three-dimensional structure of the NHL repeats is highly conserved between flies and mice. A molecular modeling study suggested that misfolding of NHL repeats in TRIM32 induced changes in the three-dimensional structure and interactions of a TRIM32 protein with its substrates or other proteins [35,51]. The NHL repeats bind to another full-length TRIM32 in vitro [52], suggesting that the NHL repeats contribute to the oligomerization of TRIM32.

### 3.3. Autoubiquitination of TRIM32

Ubiquitination, a post-translational modification, affects the subcellular localization, activity, or turnover rate of target proteins [53]. Via its E3 ubiquitin ligase activity, TRIM32 undergoes autoubiquitination and ubiquitinates other proteins [4]. Ubiquitination of skeletal muscle proteins by TRIM32 and the consequences in skeletal muscle are discussed throughout this review.

The subcellular localization of TRIM32 depends on its autoubiquitination [54,55]. In mouse brain lysates, ubiquitinated TRIM32 was found in the cytoplasmic fraction, and nonubiquitinated TRIM32 was found in the nuclear fraction [54]. In embryonic and adult mouse brains, the interaction between ubiquitinated TRIM32 and PKCζ led to TRIM32 retention in the cytosol of neural stem cells. Abrogation of the interaction between ubiquitinated TRIM32 and PKCζ allowed TRIM32 to be translocated to the nucleus, where it bound to c-Myc. The binding of TRIM32 to c-Myc in the nucleus resulted in the degradation of c-Myc due to c-Myc ubiquitination by TRIM32, which led to neuronal differentiation. In the lysates of mouse brain or skeletal muscle, 14-3-3 protein is bound to phosphorylated TRIM32 via the action of PKA [55]. In HEK293 cells, the interaction between 14-3-3 and phosphorylated TRIM32 was found to prevent the autoubiquitination of TRIM32 and the formation of TRIM32-containing cytoplasmic bodies, resulting in the localization of TRIM32 to the cytosol, where it diffused and did not accumulate in cytoplasmic bodies.

## 4. TRIM32-Binding Proteins in Skeletal Muscle

TRIM32 binds to various proteins in different types of cells [4,36]. To date, most TRIM32-binding proteins have been identified as targets of TRIM32 E3 ubiquitin ligase activity. Muscle proteins that bind to TRIM32 include actin [56], α-actinin [56,57], desmin [56], dysbindin [58], tropomyosin [56], and c-Myc [59], which are related to skeletal muscle atrophy; SERCA1a [52], which is related to Ca^2+^ uptake into the SR during skeletal muscle relaxation; protein inhibitor of activated STAT (PIASy) [60,61], N-Myc downstream-regulated gene 2 (NDRG2) [62], and c-Myc [59], which are related to skeletal muscle regeneration; p62/sequestosome1 (SQSTIM1) [32], activating molecule in BECN1-regulated autophagy protein 1 (AMBRA1) [63], and Unc-51 like autophagy activating kinase 1 (ULK1) [63], which are related to autophagy. TRIM32-binding proteins in non-muscle cells or tissues, N-Myc proto-oncogene protein (MYCN) [64], Abl-interactor 2 (Abi2) [65], p53 [66], AT-rich interaction domain 1A (ARID1A) [67], and X-linked inhibitor of apoptosis (XIAP) [68] are related to carcinogenesis; c-Myc [59], PKCζ [54], and octamer-binding transcription factor 4 (Oct4) [69] are related to differentiation; stimulator of interferon genes (STING)/MITA/TMEM173 [70], polymerase basic protein 1 (PB1) [71], and OTU domain-containing deubiquitinase with linear linkage specificity (OTULIN) [72] are related to immunity. Among these TRIM32-binding proteins, the binding of PIASy, SERCA1a, Abi2, or XIAP to TRIM32 is mediated by the NHL repeats in TRIM32 [52,61,65,68]. Five representative skeletal muscle proteins that bind to TRIM32 are described in detail below. Of the five proteins listed below, all of the modifications are related to TRIM32 ubiquitin ligase activity but the binding of SERCA1a and TRIM32 is not.

In addition to the binding of TRIM32 to proteins, TRIM32 binds to microRNAs (miRNAs) positively or negatively to regulate miRNA-mediated gene expression [73,74,75,76]. For example, TRIM32 and NHL-2 (the *Caenorhabditis elegans* ortholog of TRIM32) are enhancers of gene expression [73,74]. Mei-P26 and mLin41 are repressors of gene expression [75,76]. For more details and explanations on the implications of TRIM family regulatory functions, including those of TRIM32, on miRNAs, please refer to review articles [77,78,79].

### 4.1. Actin

Actin is a ubiquitous cytoskeletal protein and the first identified substrate of TRIM32 in skeletal muscle [80]. In skeletal muscle, actin composes thin filaments (also called actin filaments) [5]. Turnover of actin is tightly regulated during muscle development and remodeling [81]. In skeletal myotubes, α-actinin connects thin filaments to the Z-lines of sarcomeres [5].

TRIM32 promotes the ubiquitination and degradation of skeletal muscle proteins that constitute thin filaments and Z-lines in vitro; that is, TRIM32 mediates the degradation of actin and tropomyosin in thin filaments and α-actinin in the Z-line [56]. Downregulation of TRIM32 in mouse tibialis anterior (TA) muscle using short hairpin RNA (shRNA) under a fasting condition attenuated the loss of thin filaments and skeletal muscle fibers, which alleviated fasting-induced atrophy [56].

Thick filaments are composed mainly of myosin, and myosin binds to TRIM32 in vitro; however, myosin is not a target of TRIM32 E3 ubiquitin ligase activity [57], suggesting that not all proteins that bind TRIM32 are substrates of TRIM32.

### 4.2. Desmin

Desmin is a major muscle-specific protein in intermediate filaments and is located mainly at the periphery of the Z-line of sarcomeres in striated muscle cells and the dense body in smooth muscle cells [82]. Desmin plays a role in maintaining the structural integrity of thin filaments in sarcomeres [56]. In mouse TA muscle under a fasting condition, phosphorylated desmin bound to TRIM32, which triggered the ubiquitination of desmin by TRIM32, leading to the degradation of desmin and the Z-line proteins, and ultimately causing the degradation of thin filaments [56]. TRIM32 knockdown in the mouse TA muscle using shRNA under a fasting condition attenuated the degradation of desmin and thin filaments and the loss of skeletal muscle fibers [56].

### 4.3. c-Myc

**Figure 3:**
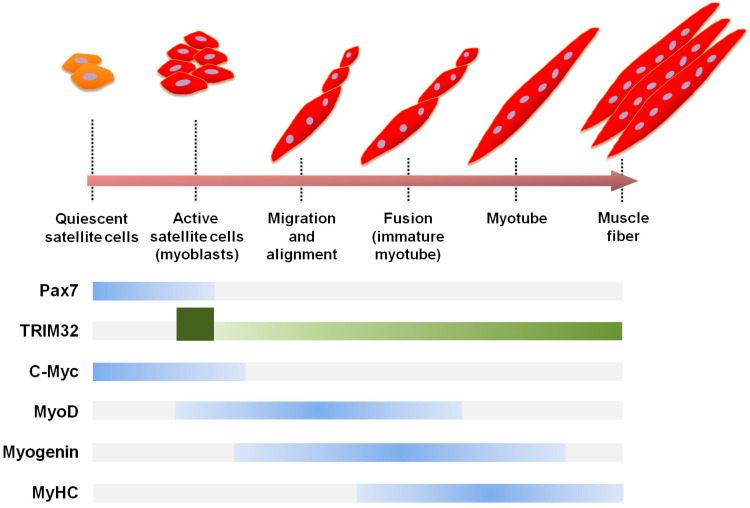
Expression patterns of cell proliferation or differentiation/myogenic factors during the terminal differentiation of myoblasts into myotubes. A darker color represents higher expression of each protein during terminal differentiation. The dark green square in TRIM32 indicates an extremely high expression level of TRIM32 compared to those in other periods of terminal differentiation.

c-Myc in skeletal muscle fibers is a cell proliferation factor that represses the expression of MyoD and myogenin, which are cell differentiation/myogenic factors [83]. TRIM32-mediated ubiquitination and degradation of c-Myc induces muscle differentiation [59]. In C2C12 myoblasts (a mouse myoblast line), TRIM32 bound to c-Myc and mediated c-Myc ubiquitination and degradation [59]. c-Myc degradation released c-Myc inhibition on MyoD and myogenin expression, which induced the terminal differentiation of C2C12 myoblasts into myotubes by stopping their proliferation (Figure 3).

### 4.4. Dysbindin

Dysregulated TRIM32 E3 ubiquitination is a pathogenic mechanism in LGMD2H patients harboring the TRIM32 genetic variants, R394H or D487N [58]. In TA muscle from guinea pigs, TRIM32 colocalized with dysbindin at the periphery of the Z-line, and TRIM32 bound and ubiquitinated dysbindin in vitro [58]. The TRIM32 R394H and D487N mutants impaired TRIM32 E3 ubiquitination of dysbindin in a heterologous expression system (COS-7 cells), and these mutants were mislocalized to the cytosol, not to cytoplasmic bodies. The R394H mutant decreased the monoubiquitination of dysbindin by wild-type TRIM32 by binding to wild-type TRIM32. The D487N mutant maintained binding ability to dysbindin, but it impaired dysbindin monoubiquitination. TRIM32 knockdown in C2C12 myoblasts using short interfering RNA (siRNA) increased the dysbindin level.

As a contributor to biogenesis of lysosome-related organelle complex 1 (BLOC-1), dysbindin regulates the maintenance and trafficking of intracellular vesicles, including lysosomes, endosomes, and autophagic vacuoles [84]. It is possible that impaired vacuolization or vacuolar trafficking due to the defective ubiquitination of dysbindin by the genetic variants of TRIM32 contributed to pathogenesis in LGMD2H patients [38,52,84,85,86,87,88]. Indeed, abnormal vacuoles or excessive cytoplasmic vacuolization are characteristics of skeletal muscles in LGMD2H patients and TRIM32-knockout mice [38,85,86,87,88]. Abnormal cytoplasmic vacuoles and tubular structures were induced in mouse primary skeletal myotubes that overexpressed a TRIM32 deletion mutant in which all the NHL repeats (where the LGMD2H-causing TRIM32 variations were clustered) were missing [52].

### 4.5. SERCA1a

SERCA1a is the protein that mediates Ca^2+^ uptake from the cytosol to the SR during skeletal muscle relaxation [17]. Binding of TRIM32 to SERCA1a regulates SERCA1a activity [52]. Via its NHL repeats, TRIM32 bound to SERCA1a in lysates of mouse primary skeletal myotubes or rabbit skeletal muscle [52]. In mouse primary skeletal myotubes, the binding of TRIM32 to SERCA1a induced an increase in the Ca^2+^ level in the SR and a reduction in the Ca^2+^ level in the cytosol with no change in the amount of SERCA1a; this could be because after binding to TRIM32, SERCA1a activity was enhanced, and TRIM32 did not mediate the degradation of SERCA1a (i.e., these proteins bound in an ubiquitin-independent manner).

It is possible that SERCAs and TRIM32 are correlated with each other via Notch signaling in skeletal muscle [89,90,91]. The Notch signaling pathway is a key regulator of the development and regeneration of skeletal muscle by maintaining the quiescence of satellite cells and promoting their self-renewal [92]. Hippocampus from TRIM32-knockout mice showed upregulated Notch-related gene expression [89]. SERCA1a is involved in Notch signaling; that is, SERCAs are enhancers of oncogenic Notch signaling, while SERCA inhibitors are Notch signaling suppressors [90,91]. Studies on the functional relevance of the Notch signaling pathway along with TRIM32 and SERCA1a activity in skeletal muscle could lead to additional perspective and greater understanding of the pathophysiological mechanisms underlying LGMD2H caused by *TRIM32* variants.

## 5. TRIM32 in Skeletal Muscle

### 5.1. Various Expression Levels of TRIM32 in Skeletal Muscle

TRIM32 in skeletal muscle gained attention almost ten years after it was identified as a Tat-binding protein [2,57]. Variations in the expression levels of TRIM32 under various in vivo conditions, as shown below, seemed to cause some difficulties in the characterization of TRIM32 in skeletal muscle.

In mice, the expression level of TRIM32 in skeletal muscles is much lower than that in other tissues or organs [38]. For example, the expression level of *TRIM32* mRNA in gastrocnemius and hamstring muscles was approximately 100-fold lower than that in the brain [38]. Furthermore, the expression level of TRIM32 protein varies from one muscle to another [57]. Higher expression of TRIM32 was found in the gastrocnemius, hamstring, and triceps than in the soleus, quadriceps, TA, or diaphragm muscles in mice [57].

TRIM32 plays various roles in skeletal muscle by being expressed throughout all stages of skeletal muscle cells, from its expression in satellite cells to that in muscle fibers of adult tissue (Figure 3) [59,93]. Specifically, TRIM32 is expressed in satellite cells (called primary myoblasts in the literature), myoblasts (proliferating satellite cells in culture), immature myotubes midway through their differentiation phase, fully differentiated mature myotubes, and muscle fibers in adult skeletal muscle tissues, but is not expressed in quiescent satellite cells.

The TRIM32 expression pattern in skeletal muscle is complex. Interestingly, the expression of *TRIM32* mRNA in mouse primary myoblasts was sixteen-fold higher than that in adult skeletal muscle tissues such as mouse gastrocnemius and hamstring muscles [93]. TRIM32 expression was low in proliferating mouse myoblasts under a culture condition, increased at the beginning of terminal differentiation, and was maintained during the terminal differentiation period (Figure 3) [60]. However, why the extremely high expression level of TRIM32 in primary myoblasts (indicated by the dark green square in Figure 3) is reduced to the basal level at the beginning of terminal differentiation and increases again during terminal differentiation is unclear. The regulatory mechanism underlying these changes in TRIM32 expression during their transition from primary myoblasts to myotubes remains to be determined.

### 5.2. TRIM32 Plays a Positive Role in Terminal Differentiation

TRIM32 is required for the terminal differentiation of myoblasts into myotubes during the regeneration of skeletal muscle at injury sites to maintain skeletal muscle mass at a physiological level [57,59,83]. As previously introduced in this review (Section 4.3), TRIM32-mediated ubiquitination and degradation of c-Myc mediated by its direct binding to c-Myc in C2C12 myoblasts releases c-Myc inhibition on MyoD and myogenin, which induces the differentiation of C2C12 myoblasts into myotubes (Figure 3) [59,83]. The expression level of TRIM32 was increased during the terminal differentiation of C2C12 myoblasts, and the overexpression of TRIM32 in C2C12 myoblasts promoted their terminal differentiation [57,59]. However, a transient overexpression of TRIM32 in mouse primary skeletal myotubes during the final two days of the terminal differentiation period did not change the width of the myotubes (a factor used to estimate the degree of terminal differentiation) or the expression level of myogenin in fully differentiated myotubes compared to those in control myotubes [52], suggesting that TRIM32 plays a positive role in terminal differentiation at injury sites over a certain period and/or only at critical time points.

## 6. TRIM32 and Limb–Girdle Muscular Dystrophies (LGMDs)

### 6.1. LGMDs

LGMDs constitute a heterogeneous group of autosomal disorders [94,95,96,97]. A systemic review of patients with muscular dystrophies worldwide over fifty-three years, from 1960 to 2013, was previously conducted [98]. Depending on the methods and factors used for analyses, LGMDs rank as the third, fourth, or fifth most prevalent type of skeletal muscular dystrophy.

In general, patients with LGMDs show defects primarily in skeletal muscle, mild-to-severe phenotypes, variable onset, and diverse clinical features, such as progressive and predominant proximal skeletal muscle weakness, degeneration predominantly in the shoulder and pelvic girdle, scapular winging, and skeletal muscle atrophy, resulting in difficulties in daily life without other people’s help or aids [35,99]. To date, the pathophysiological mechanisms underlying LGMD occurrence are not clearly understood, and unfortunately, there is no way to treat patients with LGMDs by directly alleviating the pathological symptoms.

In 2018, the classification of LGMDs was refined [100]. The refined classification of LGMDs, including the names of genes and proteins of LGMD-causing genetic variants, is presented in Table 1 [100,101,102,103]. Several LGMDs are not listed in the refined classification because they were found to be associated with other diseases, discovered in only one family and not in any patients subsequently diagnosed, or simply misreported. Newly discovered LGMDs are included in the refined classification. Genetic variations in *COL6A1*, *COL6A2*, or *COL6A3* induce both autosomal dominant (classified as LGMDD5) and recessive LGMDs (classified as LGMDR22) [103].

### 6.2. LGMD2H/LGMDR8

According to their clinical features, diseases that are caused by genetic variations in the *TRIM32* gene are classified into two groups. One group consists of Bardet–Biedl syndrome (BBS) [104,105]. The pathogenesis of BBS involves genetic variations mainly in the B-box domain of TRIM32 and is not associated with muscle involvement. Patients with BBS show a wide range of symptoms, such as retinal dystrophy, truncal obesity, learning disability, male hypogenitalism, female genitourinary malformation, polydactyly, and/or adrenal dysfunction. The other group is constituted by LGMD2H, an autosomal recessive muscular dystrophy [3,51,96,106,107,108]. LGMD2H is also known as LGMDR8 in the refined classification of LGMDs (Table 1), and, until now, LGMD2H has been used more than LGMDR8 in the literature. Patients with LGMD2H show progressive skeletal muscle weakness and atrophy, mainly proximal weakness but also facial, axial, or distal weakness.

Most TRIM32 genetic variations that cause LGMD2H in humans are point mutations, and some patients present with deletion mutations [3,38,51,107]. Point mutations are clustered mainly in the highly conserved NHL repeats of TRIM32. Interestingly, although TRIM32 is widely expressed in various tissues, patients with TRIM32 genetic variations in NHL repeats present with mainly muscular symptoms and minimal or no dysfunction in other tissues or organs (details are available in Section 6.3). This point is critical when deliberating whether TRIM32 in skeletal muscle plays different or additional roles compared with its effect on other tissues or organs. It is possible that no protein plays the exact same role as TRIM32 in skeletal muscle. Additionally, it is also possible that the muscular symptoms caused by LGMD2H are simply related to the unique property of skeletal muscle, which constitutes a larger portion of the body than any other tissue or organ.

Cellular studies on biopsy samples or primary myoblasts from LGMD2H patients revealed cellular abnormalities, such as rounded muscle fibers with an increased number of nuclei, cytoplasmic segmental vacuoles derived from the SR and t-tubules, autophagic vacuoles, Z-disc streaming, myofibrillar degeneration, atrophic fibers, a diminished satellite cell pool, and a reduced satellite cell capacity for proliferation and differentiation [85,86,87,106,109]. The latter two abnormalities are discussed in detail in a separate section of this review (8. TRIM32 and Skeletal Muscle Atrophy).

### 6.3. LGMD2H-Causing Genetic Variants of TRIM32 in Humans and Disease Symptoms

Some genetic variations in the *TRIM32* gene that cause LGMD2H in humans are introduced below. Locations of the genetic variations in the *TRIM32* gene that are described in this review are presented in Figure 1 and listed in Table 2. Among these variations, four genetic variations in NHL repeats conserved between *Drosophila* and humans are indicated in the three-dimensional structure of the NHL repeats in *Drosophila* Abba/Thin in Figure 2.

In 2002, TRIM32 D487N (c.1459G>A, a homozygous missense mutation in the third NHL repeat of TRIM32) was first identified as the cause of LGMD2H in the Hutterite population of Manitoba [3]. TRIM32 D487N is also the most frequently reported genetic mutation in TRIM32. D487 is one of the two most conserved residues in NHL repeats [3]. Patients carrying the TRIM32 D487N mutation exhibit proximal muscle weakness, mild ankle contraction, and calf pseudohypertrophy but no cardiac or respiratory symptoms [88,109]. They also show symptoms that overlap with those typical of sarcotubular myopathy (STM) [109].

TRIM32 V591M (c.1771G>A) is another representative LGMD2H-causing mutation in humans [87]. Patients with the TRIM32 V591M mutation present with various clinical features, such as ankle contraction, paravertebral muscle atrophy, foot drop with proximal upper limb weakness, and distal weakness in the lower and upper limbs but no cardiac or respiratory symptoms. Biopsy samples of the brachii muscle obtained from patients exhibited reduced expression of TRIM32 V591M mutant compared with that of TRIM32 in healthy individuals. Primary myoblasts from these patients also showed a reduction in the expression of myosin heavy chain (MyHC) and myogenin. Patients harboring the TRIM32 V591M or N217S/F568del (a double mutation near the coiled-coil region and at the NHL repeats) mutation present with many autophagic vacuoles and a profound reduction in the number of satellite cells. The bicep brachii muscle or primary myoblasts from patients with the TRIM32 N217S/F568del or C39LfsX17 (a genetic mutation in the RING domain) mutation showed reduced expression of each variant as those in patients harboring the TRIM32 V591M mutation.

A patient harboring the TRIM32 D12EfsTer44 (c.35dupA, a single-base-pair insertion in exon 2 of the *TRIM32* gene, resulting in a frameshift and premature truncation of 44 residues downstream) mutation present with a waddling gait and slowly progressive weakness of symmetric proximal lower limbs but no respiratory or cardiac symptoms [110]. In addition, this patient exhibited no weakness in the upper limbs, axial musculature, or cranial muscles. Biopsy samples from the quadriceps muscle of this patient showed myopathic features, including fibers with internalized nuclei and occasional myophagocytosis, rounded fibers of various sizes, type II fiber grouping, and atrophic fibers.

Interestingly, a patient harboring the TRIM32 D588del (c.1761_1763delGAT, a mutation in the fifth NHL repeat of TRIM32) mutation presented with much milder symptoms than patients carrying other genetic variants of TRIM32 [51]. The patient presented with muscle cramps only after exercise, scapular winging with no pseudohypertrophy of the calves, and normal muscle strength in the upper and lower limbs.

Other genetic variants in the NHL repeats of TRIM32 have also been identified, and some of these variants lead to mild abnormalities in other organs as well as muscular dystrophies in skeletal muscle [51]. Patients harboring the TRIM32 R394H (c.1180G>A) mutation presented with skeletal muscle weakness and paresthesia, showing progressive difficulties in rising from the floor, climbing stairs, and walking; atrophy in both the upper and lower limbs with muscle weakness; retraction in the ankles, knees, or Achilles tendons; scapular winging; signs of a right bundle branch block on an electrocardiogram; and a reduction in forced expiratory vital capacity [51]. A patient harboring the TRIM32 T520TfsX13 (c.1559delC) mutation presented with slowly progressing proximal weakness and skeletal muscle wasting including marked atrophy in the shoulder, abduction of the arms, and respiratory weakness [51].

Case reports on recently identified LGMD2H-causing TRIM32 genetic variants in humans have been reported, but the pathophysiological mechanisms of the clinical features attributed to these variants have not been extensively studied [85,113,115]. A patient harboring the TRIM32 P619S (c.1855C>T) mutation presented with both LGMD2H and multiple sclerosis, exhibiting muscle weakness and atrophy in all muscles of the shin along with other symptoms [115]. A patient harboring the TRIM32 S594N (c.1781G>A) mutation presented with both LGMD2H and STM, exhibiting slowly progressing weakness of the lower limb girdle muscle, severe abnormalities in the myofibrillar network, lobulated or whorled fibers, and multiple vacuoles but no cardiac or respiratory symptoms [113].

## 7. Animal Models of LGMD2H

### 7.1. A TRIM32-Knockout Mouse Model

TRIM32-knockout mice, in vivo models of human LGMD2H, are viable and fertile and share many skeletal muscle features with patients with LGMD2H [38,60,87,88,106,109,111]. Therefore, these model mice are very useful for understanding the pathological mechanisms underlying LGMD2H in humans.

TRIM32-knockout mice exhibited an increase in body weight by approximately 10% by the age of 8 weeks [38]. Interestingly, however, the muscle weight of both the fast-twitch TA and slow-twitch soleus muscles in these mice was reduced (i.e., the muscle weight/body weight ratio was reduced), indicating skeletal muscle atrophy because of TRIM32 knockout. The muscle strength of the TRIM32-knockout mice was also reduced (i.e., skeletal muscle weakness). The average peak tension for the grip strength of the forelimb and latency to fall in a wire hang test was decreased by TRIM32-knockout by approximately 17% and 1.8-fold, respectively. Skeletal muscle atrophy and weakness were identified in patients with LGMD2H, as described previously in this review [51,87,88,109,110,113,115].

The TRIM32-knockout mice presented with myopathic and dystrophic phenotypes at the cellular and tissue levels that were similar to those of skeletal muscle fibers from patients with LGMD2H [38,87,106,109]. In cross-sections of quadriceps and hamstring muscles from the TRIM32-knockout mice, the fibers were characterized with a small diameter, internalized nuclei, and fiber splitting [38]. In the soleus muscle, the amount of total fast MyHC (type IIb) was significantly decreased by TRIM32 knockout, and in contrast, the amount of slow MyHC (type I) was significantly increased, suggesting a shift toward a phenotype manifesting slower muscle fibers. In addition, TA and soleus muscles from the TRIM32-knockout mice showed disorganized sarcomeres, autophagic double-membrane vacuoles, a dilated sarcotubular system, Z-line streaming, and myofibrillar degeneration.

The TRIM32-knockout mice also acquired neurogenic phenotypes as well as myopathic and dystrophic phenotypes, as indicated by a decrease in the concentration of neurofilament proteins in the brain and a reduction in the diameters of myelinated motor axons [38]. Similar to TRIM32-knockout mice, LGMD2H patients with a frameshift (c.1560delC, C521VfsX13) or a deletion mutant of TRIM32 (30-kb intragenic deletion in the *TRIM32* gene) showed signs of both myopathy and neuropathy [111]. For example, they presented with slight type I muscle fiber dominance, a decrease in the conduction velocities of motor nerves, and abnormalities in myopathic and neurogenic electromyography of the leg muscles.

Protein extracts of quadriceps muscles excised from TRIM32-knockout mice that had been exposed to atrophy-inducing dexamethasone revealed defects in autophagic flux, as indicated by a reduced level of LC3-II (an autophagy marker protein) [63], suggesting that impaired autophagy during skeletal muscle atrophy may contribute to LGMD2H pathogenesis. In addition, similar to LGMD2H patients, TRIM32-knockout mice presented with premature senescence of satellite cells and impaired terminal differentiation of myoblasts into myotubes [38,60]. These outcomes are closely related to skeletal muscle atrophy and are discussed in detail in a separate section of this review (8. TRIM32 and Skeletal Muscle Atrophy).

### 7.2. A TRIM32 Knock-In Mouse Model

Similar to TRIM32-knockout mice, knock-in mice harboring the LGMD2H-causing TRIM32 D489N (c.1465G>A; corresponding to human D487N, c.1459G>A) mutation presented with myopathic, dystrophic, and neurogenic phenotypes [93]. Similar to LGMD2H patients harboring the TRIM32 V591M, C39LfsX17, or N217S/F568del mutations [87], a reduction in TRIM32 D487N mutant expression was identified in the knock-in mice. These reports suggest that insufficient amounts of a TRIM32 variant as well as the aforementioned mutational effects may induce LGMD2H pathogenesis in patients. This finding is supported by the prediction that mutations in NHL repeats cause conformational changes in TRIM32, which may substantially decrease protein stability and reduce the protein expression level [51].

### 7.3. Drosophila Models

TRIM32-knockout and D489N knock-in mice, as models of LGMD2H, presented with neurogenic as well as myopathic and dystrophic phenotypes [38,93], which made it difficult to interpret the phenotypes of these model mice because distinguishing the roles of TRIM32 in skeletal muscle in the model mice with the dual phenotype is not possible; nevertheless, these mouse models of LGMD2H provided an invaluable understanding of the pathological mechanisms underlying LGMD2H. In addition, TRIM32 abrogation or genetic mutation in satellite cells induced by TRIM32-knockout or D489N knock-in and in patients with LGMD2H-induced defective muscle regeneration [59,60] additively aggravated myopathic abnormalities in these mice and patients. *Drosophila* models have been studied to resolve these difficulties and concerns. Abba/Thin, the *Drosophila* ortholog of TRIM32, is encoded by the *CG15105/another b-box affiliate* (*abba*, allelic to *thin*) gene and expressed in both *Drosophila* larval and adult muscles [4,49]. The natural lack of satellite cells in *Drosophila* larval muscles provides an advantage to the use of *Drosophila* models of LGMD2H because it reveals “muscle-unique” TRIM32 functions and pathogenic effects of TRIM32 genetic variants. Clearly, a decrease in neuronal TRIM32 in *Drosophila* using siRNA did not contribute to muscle pathology as no alteration in muscle structure or function was identified [116].

Abba/Thin is expressed in the developing embryonic musculature and is located at the Z-line in larval muscles and at the M-line in adult indirect flight muscles [4,48,49,117]. A spontaneous recessive lethal mutation in *thin* gene (*lethal(2)thin*) produced long and thin pupae due to the inability of the larvae to be shortened prior to pupariation (i.e., they presented with muscle atrophy and failed muscle movement) [117]. Embryos harboring *lethal (2) thin* started to develop normally; however, the majority of the offspring showed embryonic or pupal lethality [48]. *Drosophila* with *lethal(2)thin* exhibited abnormal features in skeletal muscle, such as progressive unbundling of myofibrils and breakdown of the costamere as well as progressive muscular degeneration and abnormal sarcomere development [48], suggesting that *Drosophila* can be used as another very useful animal model of LGMD2H to understand the pathological mechanisms underlying LGMD2H.

Deletion mutations in the abba/*thin* gene, which are not evident the RING domain, B-box domain, or coiled-coil region, led to similar morphological and sarcomeric defects as those exhibited by *Drosophila* harboring *lethal(2)thin* [49]. The embryos initially formed muscles; however, F-actin and myosin striations became progressively disrupted from increased contractile forces during larval muscle growth and development. These abnormalities led to myofiber damage and atrophy, compromised muscle function, and lethality during pupation. In adult *Drosophila* muscles, Abba/Thin was also shown to be essential for maintaining the structural integrity and function of sarcomeres via its RING domain, B-box domain, coiled-coil region, and NHL repeats [49].

Myopathic and dystrophic phenotypes in skeletal muscle are also found in transgenic *Drosophila* expressing LGMD2H-causing TRIM32 R394H, D487N, or T520TfsX13 mutant in an Abba/Thin-knockout background; for example, these mutant *Drosophila* presented with a reduction in muscle size, reduced flight ability, abnormalities in myofibrils, altered nuclear morphology, reduced sarcomere structure integrity, and myofibers degeneration in adult *Drosophilae* indirect flight muscles [116]. No neurogenic abnormality was observed in the transgenic *Drosophila*, emphasizing that the myopathic and dystrophic phenotypes of patients with LGMD2H are caused by muscle defects induced by LGMD2H-causing genetic variations in TRIM32 and are not associated with neurogenic defects. Similar to the reduced expression of TRIM32 V591M, C39LfsX17, or N217S/F568del mutant in LGMD2H patients or TRIM32 D487N mutant in knock-in mice [87,93], the expression level of TRIM32 R394H, D487N, or T520TfsX13 mutant in transgenic *Drosophila* was also reduced. The expression of wild-type full-length Abba/Thin in *Drosophila* carrying genetic mutations or deletion mutations, which was induced by introducing the full *abba* gene into muscle tissue, rescued the myopathic and dystrophic phenotype in *Drosophila* models of LGMD2H [48,49]. In addition, the expression levels of two costameric proteins, βPS integrin and sarcoglycan δ, were elevated in transgenic *Drosophila* [116], suggesting the possibility that changes in the expression level of costameric proteins contribute to disease progression in LGMD2H patients.

All these studies based on *Drosophila* models of LGMD2H suggest the conservation of pathological genetic variations in the *TRIM32* gene among flies, mice, and humans. For more details about the involvement of Abba/Thin in *Drosophila* muscle development, particularly its effect on cell proliferation and death, please refer to the review article by Bawa, S. et al. [4].

## 8. TRIM32 and Skeletal Muscle Atrophy

### 8.1. Skeletal Muscle Atrophy

Atrophy involves protein degradation via the ubiquitin-proteasome system and the partial or complete loss of a part of the body [118,119]. The ubiquitin-proteasome system plays an important role in the maintenance of skeletal muscle mass [120]. Loss of skeletal muscle is called “skeletal muscle atrophy” and is induced by an increase in skeletal muscle proteins degradation relative to its synthesis [121,122]. Skeletal muscle atrophy is a result of fasting due to malnutrition or food deprivation, immobility due to disuse of skeletal muscle because of denervation, various diseases including skeletal muscle diseases, cancers, or other systemic diseases, or a sedentary lifestyle [119,123]. Skeletal muscle atrophy that develops during normal aging is called “sarcopenia” [124]. For example, in a large and heterogeneous group consisting of 468 men and women aged from 18 to 88 years, sarcopenia induced an approximate 0.25~0.4% loss in total skeletal muscle mass per year, with the amount depending on sex and age [125]. The loss of skeletal muscle aligned with a reduction in muscle function such as force production and fatigue resistance.

Skeletal muscle atrophy involves the upregulation of a group of genes called atrogins [126,127]. Atrogins are found in the ubiquitin-proteasome pathway, and they include E3 ubiquitin ligases such as muscle RING finger 1 (MuRF1) and muscle atrophy F box (MAFbx). MuRF1 is also called TRIM63 and is critical for the degradation of myofibrillar components. MAFbx is also called atrogin-1 and is critical for the disruption of muscular structures, the inhibition of myoblast differentiation and muscle regeneration, and the degradation of nonstructural proteins. The expression of MuRF1 and MAFbx is mostly upregulated in various animal models of atrophy, regardless of the cause of the atrophy [126]. Overexpression of MuRF1 in C2C12 myotubes induced cellular atrophy, and mice with either MuRF1 or MAFbx deficiency showed resistance to denervation-induced atrophy [122]. In the myofibrils of gastrocnemius muscles in mice with denervation-induced atrophy, thick filaments, but not thin filaments, were degraded by MuRF1-dependent ubiquitination [128].

Based on previous reports showing that the most obvious symptom of LGMD2H patients is skeletal muscle atrophy and that myopathic and dystrophic skeletal muscles in LGMD2H patients share features with atrophied skeletal muscles [119], studies on the pathophysiological roles of TRIM32 genetic variants in LGMD2H patients have been conducted, with particular attention paid to skeletal muscle atrophy. Notably, the role played by TRIM32 in terminal differentiation during skeletal muscle regeneration at injury sites (i.e., TRIM32 plays a positive role in skeletal muscle regeneration in response to injuries) needs to be distinguished from the role played by TRIM32 in the progression of skeletal muscle atrophy.

### 8.2. Correlation of TRIM32 with Metabolic Proteins in Skeletal Muscle Atrophy

Skeletal muscle is related to metabolism via the many glycolytic enzymes at the M- and Z-line of sarcomeres, and alterations in metabolism and muscular dystrophies are correlated with each other [12,13,14,15,16]. For example, phosphoglycerate mutase was located mainly at the M-line of sarcomeres in rat skeletal muscle fibers and in *Drosophila* flight muscle [14,15]. Enolase was located at the M-line in rabbit skeletal muscle fibers [16]. TRIM32 is required for the stability of glycolytic proteins in skeletal muscle [62]. Downregulation of enzymes associated with glycolysis/glycogenolysis was found in the gastrocnemius muscle from TRIM32-knockout mice; enzymes with downregulated expression included phosphoglucomutase-1, L-lactate dehydrogenase, pyruvate dehydrogenase, pyruvate kinase, and UTP-glucose-1-phosphate uridylyltransferase [62].

TRIM32 in conjunction with glycolytic enzymes is involved in the maintenance of muscle mass and the development of skeletal muscle [48,50,129]. Abba/Thin in *Drosophila* physically interacts with two glycolytic enzymes via its NHL repeats; these two enzymes are aldolase and phosphoglycerate mutase 78 (Pglym78, the *Drosophila* ortholog of phosphoglycerate mutase) [50]. Sarcomere disorganization was found in the Abba/Thin-deficient (*lethal(2)thin*) larval muscles of *Drosophila,* and the loss of Abba/Thin caused an approximately 50% decrease in the protein level of aldolase or Pglym78. Abba/Thin-deficient *Drosophila* also presented with narrowing and progressive degeneration of larval muscle (i.e., skeletal muscle atrophy) [48]. In addition, deficiency or mutation in the *Pglym78* gene led to embryonic muscle architecture alteration and reduced developing muscle size due to the reduced rate of myoblast fusion in *Drosophila* embryos [129]. Similarly, attenuating the expression of phosphoglycerate mutase 2 in zebrafish using morpholino-mediated knockdown resulted in thin muscles and disrupted myoblast fusion in embryos [129]. The slow and fast muscle fibers in the zebrafish were differentially affected. Large gaps were observed between syncytial fast muscle fibers, fiber width was reduced, the number of nuclei per fiber was reduced, and myoblast fusion was inefficient. In contrast, slow muscle fibers in the morphants appeared normal [129].

### 8.3. TRIM32-Mediated Autophagy in Skeletal Muscle Atrophy

Autophagy promotes cell survival by preventing cellular damage due to various cellular stresses, such as that caused by energy deficiency and cytotoxic insults [130]. Appropriate autophagy is required for maintaining muscle integrity, and the dysregulation of autophagy is involved in the progression of muscular myopathies and dystrophies with skeletal muscle atrophy [120,131]. The evidence presented below obtained from studies using various cells suggests that defects in TRIM32-mediated autophagy contribute to pathogenic atrophy in LGMD2H patients.

TRIM32 is required for the induction of autophagy under atrophic conditions to protect skeletal muscle [63,132]. The extract of quadriceps muscle from TRIM32-knockout mice with dexamethasone-induced atrophy showed defects in autophagic flux, as indicated by a reduced level of LC3-II (an autophagy marker protein) [63]. Expression of the LGMD2H-causing TRIM32 R394H or D487N mutant in TRIM32-knockdown C2.7 cells (a murine myoblast cell line) impaired autophagy induction in response to dexamethasone treatment. Impaired autophagy was also found in myoblasts that were transdifferentiated from the fibroblasts of an LGMD2H patient harboring the TRIM32 R613X (c.1837 C>T [114]) mutation.

The autophagy-promoting activity of TRIM32 in skeletal muscle is triggered via the polyubiquitination of ULK1, an upstream regulator of autophagy [63]. In 293T cells under a dexamethasone-induced atrophic condition, TRIM32 bound to ULK1 and stimulated ULK1 activity via unanchored K63-linked polyubiquitin chains [63]. The LGMD2H-causing TRIM32 R394H, D487N, D588del, T520TfsX13, C521VfsX13, or I590LfsX38 mutant [112] impaired autophagy induction in 293T cells by unbinding to ULK1 [63].

p62, an autophagy receptor that is also known as sequestosome-1 (SQSTM1), is important for sequestering autophagosome-targeted cargo and phagophore membrane scaffold development during autophagy induction [133,134]. In conjunction with p62, TRIM32 plays a dual role in autophagy by functioning as both a regulator and substrate of p62 [32]. TRIM32 binds to p62 and thus positively regulates P62 activity by mediating its monoubiquitination; conversely, p62 mediates the autophagic degradation of TRIM32 [32]. Expression of LGMD2H-causing TRIM32 D487N or V591M mutant in HEK293 cells inhibited the ability of TRIM32 to regulate p62 activity as well as its autophagic degradation [32]. These reports suggest that impaired autophagy due to impaired TRIM32-mediated regulation of p62 activity during skeletal muscle atrophy may contribute to LGMD2H pathogenesis [32,63].

### 8.4. Premature Senescence of Satellite Cells and Skeletal Muscle Atrophy in LGMD2H

Injection of cardiotoxin into the mouse TA muscle caused necrosis in many skeletal muscle fibers followed by extensive satellite cell proliferation and differentiation, which were needed to regenerate the TA muscle via compensatory actions induced after cardiotoxin insult [59]. During the regeneration of TA muscle after cardiotoxin insult, the expression of TRIM32 was increased [59]. However, during muscle reloading after hind limb suspension, TRIM32-knockout mice presented with fewer activated satellite cells in the soleus muscle, which was indicated by a reduction in the expression of Pax7 (which is specifically expressed in quiescent and newly activated satellite cells) [60]. In addition, quadriceps and hamstring muscles from TRIM32-knockout mice did not contain regenerating muscle fibers, which was indicated by the lack of development-associated MyHC expression [38].

TRIM32 is related to skeletal muscle atrophy by regulating the senescence of satellite cells [38,60]. Delayed and impaired myogenesis due to premature senescence of satellite cells in TRIM32-knockout mice was induced by the accumulation of PIASy [60,61,93]. Also known as PIASg or PIAS4, PIASy is a SUMO E3 ligase that induces senescence arrest [135]. The interaction of TRIM32 with PIASy promotes the ubiquitination and degradation of PIASy [61]. As we have repeatedly mentioned, TRIM32-knockout mice displayed skeletal muscle atrophy [38]. Primary myoblasts from the soleus muscles of the TRIM32-knockout mice showed PIASy accumulation [60]. The level of p53 (an effector of PIASy-mediated senescence) was also elevated in TRIM32-knockout primary myoblasts. Impaired degradation of PIASy led to the increased SUMOylation of cellular proteins and premature senescence of TRIM32-knockout primary myoblasts. The LGMD2H-causing TRIM32 D489N variant (which corresponds to D487N in patients with LGMD2H) expressed in mouse epidermal keratinocyte cells or evaluated via in vitro ubiquitination assays failed to bind to PIASy, to ubiquitinate PIASy or to undergo autoubiquitination [61]. Similar to the effect of PIASy, excess NDRG2 (an inhibitor of cell proliferation [136]) mediated skeletal muscle atrophy [62,137]. NDRG2 accumulated in the gastrocnemius muscles of TRIM32-knockout mice due to the absence of TRIM32-mediated ubiquitination and degradation of NDRG2 [62]. NDRG2 levels were also increased in primary myoblasts from TRIM32-knockout mice. NDRG2 overexpression in C2C12 myoblasts led to a reduction in the proliferation of C2C12 myoblasts and a delay in cell cycle withdrawal during differentiation [62]. NDRG2 gene expression in C2C12 myotubes was upregulated by atrophy-causing dexamethasone or TNFα [137].

On the other hand, the expression of TRIM32 in satellite cells from TRIM32-knockout mice was not excessively high compared to that in adult skeletal muscle tissues [93]. TRIM32 genetic variations were expressed at reduced levels in LGMD2H patients and various animal models of LGMD2H [87,93,116].

**Figure 4:**
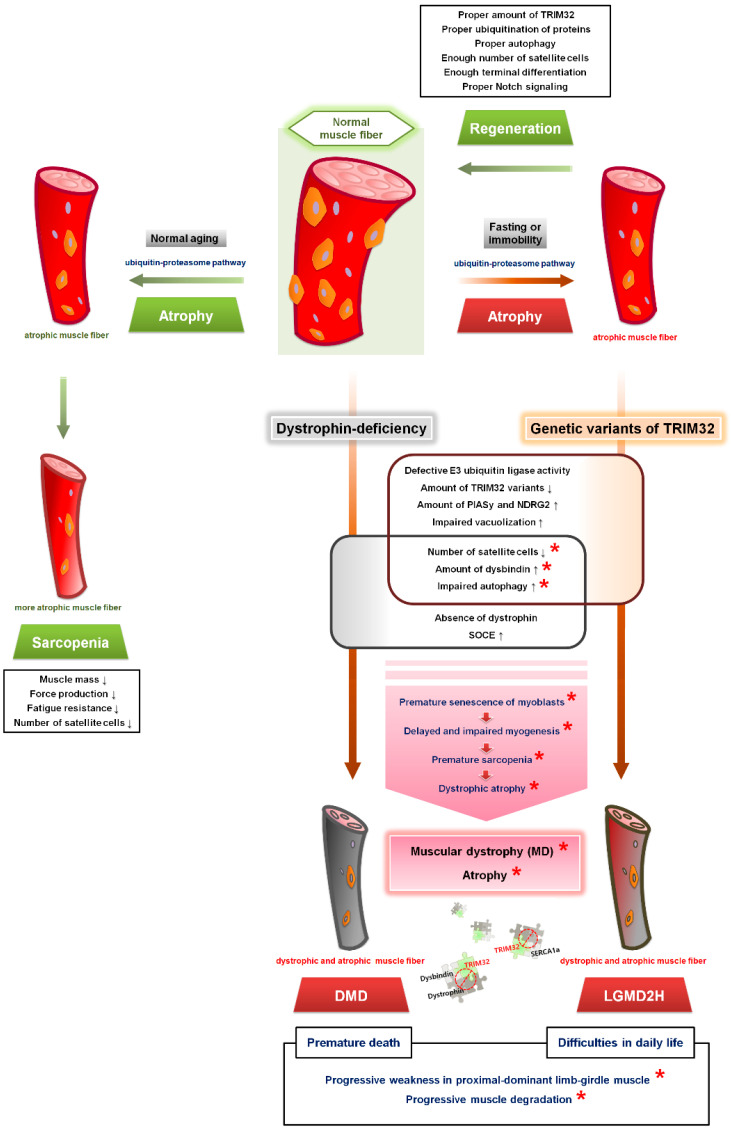
The proposed pathological mechanism of LGMD2H that is caused by TRIM32 genetic variants and correlations between LGMD2H and DMD. Pathways with green arrows and name tags indicate physiological processes, and pathways with red arrows and name tags indicate pathological processes. Asterisks in red indicate phenomena common to patients with LGMD2H and DMD. PIASy, protein inhibitor of activated STAT; SOCE, store-operated Ca^2+^ entry; MD, muscular dystrophy; DMD, Duchenne muscular dystrophy; LGMD2H, limb–girdle muscular dystrophy type 2H.

From the perspective of the premature senescence of satellite cells caused by the absence of TRIM32 in TRIM32-knockout mice, the pathological mechanism underlying LGMD2H pathogenesis induced by TRIM32 variants can be explained as follows: (1) the amount of TRIM32 variants in satellite cells/primary myoblasts is less than that in wild-type controls (i.e., the total quantity of TRIM32 variants is low); (2) the low levels of TRIM32 variants and their functional deficits cause reduced E3 ubiquitin ligase activity, which induces premature senescence of satellite cells because PIASy and NDRG2 are not ubiquitinated and thus accumulate; (3) the premature senescence of satellite cells leads to the inefficient production of the satellite cells necessary for terminal differentiation during the regeneration of skeletal muscle during/after atrophy, resulting in delayed and impaired myogenesis; (4) in parallel with the premature senescence of satellite cells, autophagy induction is reduced due to the low levels of TRIM32 variants and their functional deficits; and (5) delayed or impaired myogenesis and defective autophagy ultimately induce the acquisition of permanent atrophic, myopathic, and dystrophic phenotypes. These proposed pathophysiological roles of LGMD2H-causing TRIM32 variants are summarized in Figure 4.

Contradictory to the positive roles played by TRIM32 in the maintenance of satellite cell the senescence during terminal differentiation and regeneration of skeletal muscle at injury sites and in autophagy induction under atrophic conditions that protects muscle under various stresses [56,57,138], knockdown of TRIM32 in the mouse TA muscle using shRNA attenuated skeletal muscle wasting and loss of thin filament components during fasting [56]. Similarly, TRIM32 knockdown using shRNA in the TA muscle of mice with fasting-induced atrophy induced the growth of skeletal muscle fibers by enhancing PI3K-Akt-FoxO pathway activation [138]. During muscle unloading of the mouse hind limb via suspension (an atrophic condition), atrophy was induced, and the expression level of TRIM32 in the lysate of the medial gastrocnemius was elevated approximately twofold [57]. These reports suggest that TRIM32 may function as a atrophy-promoting protein when the satellite cell pool is not large enough and/or atrophy-inducing conditions exceed TRIM32 proautophagic capacity.

An interesting finding in a study on atrophy in TRIM32-knockout mice is worth mentioning [38,60]. In contrast to TRIM32-knockout mice with atrophy under normal feeding conditions (a single insult mediated by TRIM32-knockout) [38], surprisingly, “fasting-induced skeletal muscle atrophy” in TRIM32-knockout mice (i.e., double insults induced by both fasting and TRIM32 knockout) led to the same degree of body weight and muscle mass losses as those in wild-type mice [60]. Under fasting conditions, the ratio of myosin-to-actin or actin-to-desmin in primary myotubes obtained from the atrophic TRIM32-knockout mice was indistinguishable from that in myotubes obtained from atrophic wild-type mice, suggesting that, under both TRIM32 knockout and fasting conditions, the atrophy induced by each insult did not lead to an additive effect.

## 9. TRIM32 and DMD

### 9.1. DMD

Deficiency in the dystrophin protein due to the *DMD* gene variants in skeletal and cardiac muscles induces DMD, a recessive X-linked inherited disorder affecting approximately one in every 3500 males [139]. DMD involves progressive weakness and rapid degeneration of muscle and ultimately causes premature death. A reduction in the amount or size of the dystrophin protein induces Becker muscular dystrophy (BMD), which causes symptoms that are similar to those in DMD patients but less severe [139]. Based on a systemic review of patients with muscular dystrophies from 1960 to 2013 across the world, dystrophin-related muscular dystrophies were found to be the second most prevalent type of muscular dystrophy [98]. More recently, a clinical and genetic neuromuscular cohort analysis on Lebanese patients performed from 1999 to 2019 showed that dystrophin-related muscular dystrophies are also the second most frequently diagnosed inherited neuromuscular disorders associated with the highest number of variants [140].

### 9.2. TRIM32 in DMD

Some pathogenic factors are common to both DMD (and BMD) and LGMD2H. For example, the number of available satellite cells is low in patients with either condition. Early studies on DMD suggested that the number of satellite cells in muscle biopsy samples from DMD patients or muscle fibers from *mdx* mice (mouse models of DMD harboring a naturally occurring null mutation in the *DMD* gene) was elevated [38,60,141,142,143,144,145,146,147,148]. However, the satellite cells in the DMD samples, although high, were dysfunctional and did not participate in skeletal muscle regeneration, contributing to DMD progression [142,147]. The satellite cells in the *mdx* mice lost cell polarity due to impaired asymmetric cell division and other mitotic defects, resulting in dysfunctional satellite cells [142,147,148]. In addition, mice lacking both dystrophin and MyoD (*mdx/MyoD* double-knockout mice) or both dystrophin and the RNA component of telomerase (*mdx/mTR* double-knockout mice) presented with exacerbated muscular dystrophies compared with *mdx* mice due to a reduction in the number of functional satellite cells [145,146]. As previously mentioned in this review, the number of satellite cells from TRIM32-knockout mice was low due to premature senescence [38,60]. Because the number of available functional satellite cells are low in both conditions, DMD (and BMD) and LGMD2H are correlated with each other, and very possibly pathophysiological mechanisms may be at least partially identical (indicated by red asterisks in Figure 4).

The second common pathogenic factor in both DMD (and BMD) and LGMD2H is impaired autophagy [63,114,120,149,150]. In normal muscle, defective organelles and proteins are removed by autophagy, but impaired autophagy leads to the accumulation of dysfunctional organelles and proteins [120,149]. Autophagy is profoundly impaired in TA and diaphragm muscles from DMD patients or *mdx* mice, and the damaged organelles accumulate due to the persistent activation of Akt, a potent autophagy inhibitor [150]. Similarly, impaired autophagy has been identified in TRIM32-knockdown C2.7 cells expressing LGMD2H-causing TRIM32 R394H or D487N mutants after dexamethasone treatment to induce atrophy and in transdifferentiated myoblasts from the fibroblasts of an LGMD2H patient harboring the TRIM32 R613X mutation [63,114].

Third, elevated dysbindin is a common pathogenic factor in both the DMD (and BMD) and LGMD2H contexts [58,87,151,152,153]. Knockdown of TRIM32 in C2C12 myoblasts using siRNA resulted in elevated levels of dysbindin [58]. LGMD2H-causing TRIM32 R394H and D487N mutants showed impaired E3 ubiquitin ligase activity toward dysbindin in COS-7 cells [58]. Dysbindin is a member of the dystrophin-associated protein complex, and the dysbindin level was increased in the TA muscle of *mdx* mice [151]. Upregulation of dysbindin expression at the mRNA level was observed in muscle biopsy samples from DMD patients compared with samples from healthy individuals [152]. In biopsy samples of the femoral quadriceps muscle from patients with DMD or BMD, TRIM32 expression was downregulated in necrotic muscle fibers compared with regenerating muscle fibers [153].

Fourth, according to six different patterns of skeletal muscle weakness observed in patients with genetic skeletal muscle diseases, patients with DMD (and BMD) and LGMD share a common pattern of weakness in proximal-predominant limb girdle muscles [100]. These reports strongly suggest that DMD (and BMD) and LGMD2H share pathological mechanisms and that common molecules or pathways may be targeted to alleviate the symptoms of DMD (and BMD) and LGMD2H in patients.

TRIM32 is related to the pathogenic increase in SOCE in muscle fibers from patients with DMD and in *mdx* mice (for details on SOCE, see Section 2.3 in this review) [52,153,154,155]. Primary myotubes from patients with DMD showed an increase in SOCE [154]. The flexor digitorum brevis muscle from *mdx* mice showed excessive SOCE [155]. In biopsy samples of femoral quadriceps muscle from patients with DMD or BMD, TRIM32 expression was upregulated in regenerating muscle fibers compared with necrotic muscle fibers [153]. Mouse primary skeletal myotubes that overexpressed TRIM32 showed an increase in SOCE [52]. However, it remains to be elucidated whether SOCE is increased in muscle biopsy samples or primary myoblasts from LGMD2H patients.

## 10. TRIM32 in Non-Muscle Cells or Tissues or Other Organs

TRIM32 contributes to the development and maintenance of the brain [156,157,158]. TRIM32-knockout mice showed behaviors similar to those associated with autism spectrum disorders due to the decreased numbers of interneurons in the telencephalon and impaired generation of pyramidal neurons in the developing cerebral cortex [156,157]. Dysfunctional motor balance and coordination were identified in middle-aged TRIM32-knockout mice and were caused by decreases in the extent of dendritic arborization, dendritic spine density of Purkinje cells, and number of synapses between parallel fibers and Purkinje cells or between climbing fibers and Purkinje cells [158]. It is possible that, after long periods, the myopathic and dystrophic symptoms of LGMD2H patients can be worsened as the skeletal muscles are increasingly unused because of abnormal cerebellar motor functions, although neurogenic phenotypes are not directly related to the myopathic and dystrophic symptoms in LGMD2H patients [38,111,116]. Interestingly, the expression level of TRIM32 was increased in the occipital lobes of patients with Alzheimer’s disease [159]. For more details on the roles played by TRIM32 in the brain and their implications, please refer to other articles [74,89,160,161,162,163,164].

TRIM32-related atrophy in cardiac muscle contributes to the normal function and maintenance of cardiac muscle [165]. In mouse hypertrophic hearts induced by aortic banding, TRIM32 plays a cardioprotective role by alleviating pathological cardiac hypertrophy by interrupting Akt-dependent signaling pathways [165].

TRIM32 functions as both an oncogenic protein and a tumor suppressor [166]. As an oncogenic protein, TRIM32 contributes to cell survival during carcinogenesis by blocking UVB-induced TNFα apoptotic signaling [166]. Expression of TRIM32 at the mRNA or protein level was elevated in skin cancer cells, such as keratinocytes from a mouse epidermal carcinogenesis model, cells of squamous cell carcinoma induced by ultraviolet B (UVB), cells from chemically induced mouse papilloma, and cells of human head and neck squamous cell carcinomas [166]. In contrast, as a tumor suppressor, TRIM32 contributes to the death of different types of cancer cells, such as breast cancer, pancreatic cancer, lung cancer, gastric cancer, and colorectal cancer cells [4]. For details on the roles played by TRIM32 in carcinogenesis and their implications, please refer to other review articles [4,36,167].

Viral infections are detected by the innate immune system, and TRIM32 regulates innate immunity in response to both RNA and DNA virus infection by catalyzing the formation of the K63-linked ubiquitination of STING (also called MITA or TMEM173) [70]. TRIM32 interacts with STING and markedly enhances STING-mediated induction of IFN (interferon)-β and other innate antiviral responses. Overexpressed TRIM32 in HEK293 cells potentiated virus-triggered IFN-β expression and cellular antiviral responses. Similar to TRIM32, TRIM56, which functions as both an E3 ubiquitin ligase and deubiquitinase, catalyzes the formation of the K63-linked ubiquitination of STING and mediates IFN-1β induction during the innate immune response [168]. These reports suggest the possibility that, similar to TRIM56 replacing TRIM32 in the STING-mediated response to viral infection and inflammation, other TRIM proteins may compensate for abrogated TRIM32 or TRIM32 genetic mutations in muscles and other tissues.

## 11. Points to Be Considered When Studying TRIM32 in Skeletal Muscle

The physiological roles of TRIM32 in skeletal muscle and the pathophysiological roles of TRIM32 genetic variants in LGMD2H patients are better understood “in context”, as outlined below.

First, degrees of TRIM32-null effects vary. What kinds of skeletal muscle are used for studies? Knockout or knockdown of TRIM32 in gastrocnemius muscle, which typically shows high TRIM32 expression, may induce a more severe degree of TRIM32-null effects compared with those in TA muscle, in which TRIM32 expression is typically low.

Second, stages of skeletal muscle cells vary; i.e., quiescent satellite cells, activated satellite cells (i.e., primary myoblasts), myoblasts, immature myotubes, fully differentiated myotubes, or muscle fibers from skeletal muscle tissues differ. Skeletal muscle undergoes terminal differentiation, and skeletal muscle cells at different stages of terminal differentiation express different ratios of muscle proteins. For example, RyR1 (which is the main protein involved in Ca^2+^ release during skeletal muscle contraction) starts to be expressed in the middle stage and is expressed at the level through the last stage of terminal differentiation [169], and therefore different amounts of Ca^2+^ are released from the SR into the cytosol in cells in different differentiation stages, which results in different skeletal muscle strength.

Third, interpretations of data vary. Because skeletal muscle fibers are classified into three types on the basis of their ATP production and shortening rates, and skeletal muscle tissues are composed of mixed muscle fibers with different percentages of muscle fibers, studies with the whole extract of skeletal muscle tissues may produce different results than those using each type of muscle fiber.

Fourth, the severity of atrophy varies. How is atrophy induced in animal models? Denervation or suspension of a specific portion of the body (which induces atrophy mainly in the designated portion, for example, hind limb suspension), fasting (which induces whole-body atrophy, including atrophy in skeletal muscle and other organs), or drug treatment (which usually involves other deleterious side effects, for example, cardiotoxin or dexamethasone) induce very different degrees of atrophy severity.

## 12. Conclusions and Future Directions

To date, it has been established that TRIM32 is required for the homeostasis of skeletal muscle mass, as confirmed through three perspectives on the basis of its E3 ubiquitin ligase activity. (1) TRIM32 positively regulates the senescence of satellite cells to maintain an adequate satellite cell pool for skeletal muscle regeneration during/after atrophy. (2) TRIM32 positively regulates the terminal differentiation of myoblasts into myotubes during the regeneration of skeletal muscle at injury sites. (3) TRIM32 is a proautophagic protein that protects skeletal muscle from cellular damage under various stress conditions. In patients harboring LGMD2H-causing TRIM32 variants, defects in the E3 ubiquitin ligase activity of TRIM32 induce premature senescence of satellite cells, insufficient terminal differentiation, and defective autophagy, which results in delayed or impaired myogenesis, skeletal muscle atrophy, various muscle symptoms, ultimately causing difficulties in daily life without other people’s help or aid. More detailed mechanisms explaining these effects and the critical time points in this series of processes remain to be elucidated.

Previous studies on TRIM32 and LGMD2H-causing TRIM32 genetic variants in skeletal muscle have focused mainly on atrophy and atrophy-related protein-protein interactions, especially interactions between TRIM32 and substrates of TRIM32 functioning as an E3 ubiquitin ligase. Considering skeletal muscle functions, i.e., contraction and relaxation, studies on the roles of TRIM32 in conjunction with “functional proteins” in skeletal muscle, such as those involved in Ca^2+^ movement or handling during skeletal muscle contraction or relaxation (i.e., RyR1, DHPR, SERCA1a, or calsequestrin), in conjunction with “other well-known muscular dystrophies or diseases” such as DMD, BMD, or malignant hyperthermia, may provide additional critical clues for understanding the physiological roles of TRIM32 and the pathophysiological mechanisms of LGMD2H-causing TRIM32 genetic variants in patients.

## Figures and Tables

**Table 1:** The refined classification of LGMDs. Several LGMDs in the classical classification are not listed in the refined classification because they were found to be associated with other diseases, found in only one family and not in other patients, or simply misreported (indicated by “none” in the refined classification) [100,101,102,103].

Autosomal Dominant LGMDs			
Refined Classification	Classical Classification	Gene Name	Protein Name (Reasons for the Exclusion)
None	LGMD1A	*MYOT*	Myotilin; excluded (myofibrillar myopathy, distal weakness)
None	LGMD1B	*LMNA*	Lamin A/C; excluded (Emery–Dreifuss muscular dystrophy, high risk of cardiac arrhythmia)
None	LGMD1C	*CAV3*	Caveolin 3; excluded (rippling muscle disease and myalgia)
LGMDD1	LGMD1D	*DNAJB6*	DnaJ heat shock protein family (Hsp40) member B6
None	LGMD1E	*DES*	Desmin; excluded (false linkage, distal weakness and cardiomyopathy)
LGMDD2	LGMD1F	*TNPO3*	Transportin 3
LGMDD3	LGMD1G	*HNRNPDL*	Heterogeneous nuclear ribonucleoprotein D-like protein
None	LGMD1H	*unknown*	Unknown; excluded (false linkage)
LGMDD4	LGMD1I	*CAPN3*	Calpain 3
LGMDD5	None	*COL6A1*, *COL6A2*, *or* *COL6A3*	Collagen type VI α1, α2, or α3 chain
**Autosomal Recessive LGMDs**			
**Refined Classification**	**Classical Classification**	**Gene Name**	**Protein Name (Reasons for the Exclusion)**
LGMDR1	LGMD2A	*CAPN3*	Calpain 3
LGMDR2	LGMD2B	*DYSF*	Dysferlin
LGMDR3	LGMD2D	*SGCA*	α-Sarcoglycan
LGMDR4	LGMD2E	*SGCB*	β-Sarcoglycan
LGMDR5	LGMD2C	*SGCG*	γ-Sarcoglycan
LGMDR6	LGMD2F	*SGCD*	δ-Sarcoglycan
LGMDR7	LGMD2G	*TCAP*	Telethonin
LGMDR8	LGMD2H	*TRIM32*	Tripartite motif-containing 32 (TRIM32)
LGMDR9	LGMD2I	*FKRP*	Fukutin-related protein
LGMDR10	LGMD2J	*TTN*	Titin
LGMDR11	LGMD2K	*POMT1*	Protein O-mannosyl-transferase 1
LGMDR12	LGMD2L	*ANO5*	Anoctamin 5
LGMDR13	LGMD2M	*FKTN/FCMD*	Fukutin
LGMDR14	LGMD2N	*POMT2*	Protein O-mannosyl-transferase 2
LGMDR15	LGMD2O	*POMGnT1*	Protein O-linked mannose N-acetylglucosaminyl transferase 1
LGMDR16	LGMD2P	*DAG1*	Dystroglycan 1
LGMDR17	LGMD2Q	*PLEC*	Plectin
None	LGMD2R	*DES*	Desmin; excluded (myofibrillar myopathy, distal weakness)
LGMDR18	LGMD2S	*TRAPPC11*	Trafficking protein particle complex 11
LGMDR19	LGMD2T	*GMPPB*	GDP-mannose pyrophosphorylase B
LGMDR20	LGMD2U	*CRPPA/ISPD*	CDP-L-ribitol pyrophosphorylase A
None	LGMD2V	*GAA*	Acid α -glucosidase; excluded (Pompe disease)
None	LGMD2W	*PINCH2*	Particularly interesting Cys-His-rich protein 2; excluded (reported in only one family)
None	LGMD2Y	*TORIAIP1*	Torsin 1A interacting protein 1; excluded (reported in only one family)
LGMDR21	LGMD2Z	*POGLUT1*	Protein O-glucosyltransferase 1
LGMDR22	None	*COL6A1*, *COL6A2*, *or* *COL6A3*	Collagen type VI α1, α2, or α3 chain
LGMDR23	None	*LAMA2*	Laminin subunit α2
LGMDR24	None	*POMGNT2*	Protein O-linked mannose N-acetylglucosaminyl transferase 2
LGMDR25	LGMD2X	*BVES*	Blood vessel epicardial substance
LGMDR (number pending)	None	*PYROXD1*	Pyridine nucleotide-disulfide oxidoreductase domain-containing protein 1

**Table 2:** List of TRIM32 genetic variants that cause LGMD2H in humans and that are discussed in this review.

Variation in DNA	Heredity	Variant Name (Protein)	Position of Mutation	Phenotype	Reference Article
35dupA	Homozygous	D12EfsTer44	Intervening region before the RING domain	LGMD2H	[110]
115_116insT	Homozygous	C39LfsX17	RING domain	LGMD2H	[87]
650A>G, 1701_1703del	Heterozygous	N217S/F568del	Intervening region between coiled-coil region and NHL repeats, 4th NHL repeat	LGMD2H	[87]
1180G>A	Homozygous	R394H	2th NHL repeat	LGMD2H	[51]
1459G>A	Homozygous	D487N	3th NHL repeat	LGMD2H	[3]
1559delC	Homozygous	T520TfsX13	5th NHL repeat	LGMD2H	[51]
1560delC	Heterozygous	C521VfsX13	5th NHL repeat	LGMD2H	[111]
1761_1763delGAT	Heterozygous	D588del	5th NHL repeat	LGMD2H	[51]
1753_1766dup14	Homozygous	I590LfsX38	5th NHL repeat	LGMD2H	[112]
1771G>A	Homozygous	V591M	5th NHL repeat	LGMD2H	[87]
1781G>A	Homozygous	S594N	5th NHL repeat	LGMD2H	[113]
1837C>T	Heterozygous	R613X	6th NHL repeat	LGMD2H	[114]
1855C>T	Homozygous	P619S	6th NHL repeat	LGMD2H	[115]

## Data Availability

Not applicable.
